# Network Pharmacology-Based Strategy Reveals the Effects of *Hedysarum multijugum* Maxim.-*Radix Salviae* Compound on Oxidative Capacity and Cardiomyocyte Apoptosis in Rats with Diabetic Cardiomyopathy

**DOI:** 10.1155/2020/8260703

**Published:** 2020-10-17

**Authors:** Shiying Zhang, Zhiying Yuan, Huaying Wu, Weiqing Li, Liang Li, Huiyong Huang

**Affiliations:** ^1^Hunan University of Chinese Medicine, Changsha, China; ^2^Department of Traditional Chinese Medicine, Shenzhen Luohu People's Hospital, Shenzhen, China; ^3^Department of Traditional Chinese Medicine, The Third Affiliated Hospital of Shenzhen University, Shenzhen, China; ^4^Department of Traditional Chinese Medicine, Shenzhen Luohu Hospital Group, Shenzhen Luohu People's Hospital, Shenzhen, China

## Abstract

**Objective:**

To explore the effects of the *Hedysarum multijugum* Maxim.-*Radix Salviae* compound (Huangqi-Danshen Compound (HDC)) on oxidative capacity and cardiomyocyte apoptosis in rats with diabetic cardiomyopathy by a network pharmacology-based strategy.

**Methods:**

Traditional Chinese Medicine (TCM)@Taiwan, TCM Systems Pharmacology Database and Analysis Platform (TCMSP), TCM Integrated Database (TCMID), and High-Performance Liquid Chromatography (HPLC) technology were used to obtain and screen HDC's active components, and the PharmMapper database was used to predict HDC human target protein targets. The DCM genes were collected from the GeneCards and OMIM databases, and the network was constructed and analyzed by Cytoscape 3.7.1 and the Database for Annotation, Visualization, and Integrated Discovery (DAVID). Finally, HDC was used to intervene in diabetic cardiomyopathy (DCM) model rats, and important biological processes and signaling pathways were verified using techniques such as immunohistochemistry.

**Results:**

A total of 176 of HDC's active components and 442 potential targets were obtained. The results of network analysis show that HDC can regulate DCM-related biological processes (such as negative regulation of the apoptotic process, response to hypoxia, the steroid hormone-mediated signaling pathway, cellular iron ion homeostasis, and positive regulation of phosphatidylinositol 3-kinase signaling) and signaling pathways (such as the HIF-1 signaling pathway, the estrogen signaling pathway, insulin resistance, the PPAR signaling pathway, the VEGF signaling pathway, and the PI3K-Akt signaling pathway). Animal experiments show that HDC can reduce fasting plasma glucose (FPG), HbA1c, and malondialdehyde (MDA) and increase superoxide dismutase (SOD) and glutathione peroxidase (GSH-Px) (*P* < 0.05). The results of immunohistochemistry showed that HDC can regulate the protein expression of apoptosis-related signaling pathways in DCM rats (*P* < 0.05).

**Conclusion:**

It was initially revealed that HDC improves DCM through its antiapoptotic and anti-inflammatory effects. HDC may play a therapeutic role by improving cardiomyocyte apoptosis in DCM rats.

## 1. Introduction

Diabetes is a very common endocrine disease and one of the most common chronic diseases in almost all countries [1]. Epidemiological studies show that the number of diabetic patients is increasing year by year. According to a study by the International Diabetes Federation, the number of diabetic patients has reached 415 million by 2015, and it is estimated that the number of diabetic patients will reach 642 million by 2040 [2]. Cardiovascular complications are an important cause of death in patients with diabetes [3, 4]. Diabetic cardiomyopathy (DCM) is one of the major cardiovascular complications of diabetes, which is characterized by changes in the structure and function of the myocardium except for other confounding factors such as coronary disease or hypertension [5]. It is estimated that approximately 12% of diabetics will experience heart failure or death to varying degrees [6]. The main mechanism of cardiovascular complications of diabetes is related to the hyperglycemia state and the accumulation of advanced glycation end products, which eventually lead to increased endothelial dysfunction, fibrosis, inflammation, and oxidative stress [5, 7]. A large number of studies have confirmed that inflammation and oxidative stress play an extremely important role in cardiovascular damage caused by high glucose [5].

At present, the therapeutic drugs for DCM are diabetes drugs (such as glucagon-like peptide-1 receptor agonist and dipeptidyl peptidase 4 inhibitor) [8], vascular therapeutic drugs (such as *β*-adrenergic receptor blockers) [9], lipid-lowering drugs (such as statins) [10], and metabolic regulators (trimetazidine) [11]. However, due to the adverse reactions of these drugs, patients' compliance has been reduced; patients also face high prices and high medical cost burden [12]. Due to the complexity and diversity of DCM, researchers have sought a variety of DCM treatments. Therefore, natural products and their derivatives are considered to be one of the sources of drugs with the most potential [13, 14]. At present, more and more preclinical studies have shown that some Chinese herbal medicines and their extracts are promising DCM therapeutic drugs, such as *Radix Salviae*, *Hedysarum multijugum* Maxim., and *Panax ginseng* C. A. Mey. [15–17]. The *Hedysarum multijugum* Maxim.-*Radix Salviae* Compound (Huangqi-Danshen Compound, HDC) is a prescription that has been used by the First Affiliated Hospital of Hunan University of Chinese Medicine for more than several decades. It is a combination and modification of Shengmai San and Tianwang Buxin Dan, which consists of *licorice* (6 g), *Hedysarum multijugum* Maxim. (20 g), *Santalum album* L. (2 g), *Aurantii Fructus Immaturus* (10 g), *Platycladi Semen* (10 g), *Radix Salviae* (15 g), *Trichosanthis Radix* (12 g), *hirudo* (3 g), *Folium Nelumbinis* (15 g), *Rehmanniae Radix Praeparata* (15 g), *Panax ginseng* C. A. Mey. (9 g), and *Poria cocos* (Schw.) Wolf (8 g). In our previous experimental studies, we found that HDC can significantly improve the glucose and lipid metabolism disorders in rats with type 2 diabetic myocardial injury and significantly reduce myocardial fibrosis and myocardial hypertrophy [18, 19]. However, its mechanism for treating DCM still needs to be further revealed and discovered.

The network pharmacology strategy provides new opportunities for revealing complex traditional Chinese medicine prescriptions [20–24]. Traditional Chinese Medicine (TCM) treats diseases following a holistic view. Network pharmacology is an important part of systematic biology. Its holistic, systemic, and drug-oriented interactions are in line with the basic characteristics of TCM, which is a new discipline that reveals the regulatory effects of compound drugs on the biological network of the body from a system level, and has established a bridge for studying the relationship between TCM and modern pharmacology [20–25]. Therefore, this study hopes to explore the molecular network of HDC intervention in DCM through a network pharmacology strategy. At the same time, GK rats with more human pathogenic characteristics were used to construct a DCM model to observe the effects of HDC on oxidative stress-related indicators and myocardial apoptosis-related proteins Bax and Bcl-2 in order to verify and supplement the mechanism of HDC treatment of DCM.

## 2. Materials and Methods

### 2.1. High-Performance Liquid Chromatography (HPLC) for HDC

#### 2.1.1. Reagent Preparation

High-Performance Liquid Chromatograph (type: e2695) was purchased from Waters Corporation. YMC-Pack Pro C18 Column (4.6 mm × 250 mm, 5 *μ*m) was purchased from YMC Inc. Astragaloside IV reference (batch number: 110766-201520), rosmarinic acid reference (batch number: 110867-201607), and salvianolic acid B reference (batch number: 111562-201716) were purchased from China Food and Drug Research Institute.

*(1) HDC Solution*. *Licorice* (6 g), *Hedysarum multijugum* Maxim. (20 g), *Santalum album* L. (2 g), *Aurantii Fructus Immaturus* (10 g), *Platycladi Semen* (10 g), *Radix Salviae* (15 g), *Trichosanthis Radix* (12 g), *hirudo* (3 g), *Folium Nelumbinis* (15 g), *Rehmanniae Radix Praeparata* (15 g), *Panax ginseng* C. A. Mey. (9 g), and *Poria cocos* (Schw.) Wolf (8 g) were purchased from the First Affiliated Hospital of Hunan University of Chinese Medicine and were identified by Professor Xia Xinhua, School of Pharmacy, Hunan University of Traditional Chinese Medicine. The herbal mixture was decocted in boiling water for 45 minutes, concentrated, and dried under vacuum to form a paste. The HDC paste was concentrated to 4 g of crude drug/mL and stored at 4°C.

#### 2.1.2. HPLC Condition

*(1) Column*. This study used the YMC-Pack Pro C18 column (4.6 mm × 250 mm, 5 *μ*m) (detection wave length: 270 nm; column temperature: 30°C; flow rate: 1.0 mL/min; mobile phase: 0.5% formic acid water (A)-acetonitrile (B); ladder washing process: 0-10 min—90% A; 10-20 min—90%-80% A; 20-40 min—80%-70% A; 40-45 min—70%-65% A; 45-55 min—65%-58% A; 55-75 min—58%-45% A; 75-85 min—45%-35% A; 85-92 min—35%-30% A; 92-102 min—30%-20% A; 102-110 min—20%-0% A; and 110-120 min—0% A).

**Figure 1:**
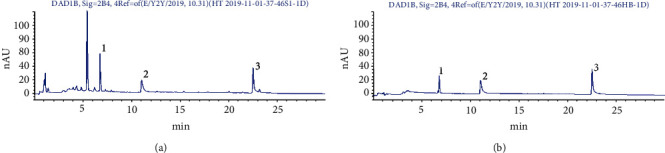
HDC fingerprint ((a) reference solution and (b) HDC solution; (1) astragaloside IV, (2) rosmarinic acid, and (3) salvianolic acid B).

The results of HPLC showed that HDC contains astragaloside IV 0.51 mg/g, rosmarinic acid 1.69 mg/g, and salvianolic acid B 2.40 mg/g. (Figure 1).

### 2.2. HDC Bioactive Compound Prediction

The TCM Systems Pharmacology Database and Analysis Platform (TCMSP) (http://lsp.nwu.edu.cn/) [26], TCM Integrated Database (TCMID) (http://www.megabionet.org/tcmid/) [27], and TCM@Taiwan (http://tcm.cmu.edu.tw/zh-tw/) [28] are used to collect all compounds in HDC and predict the pharmacokinetic properties (ADME, that is, absorption, distribution, metabolism, and excretion) of the compound molecules. Then, three parameters of ADME (drug-likeness (DL), Caco-2 permeability, and oral bioavailability (OB)) were used to predict the bioactive compounds [29–32]. Eventually, compounds with DL ≥ 0.18, Caco − 2 > −0.4, and OB ≥ 30% are considered to be bioavailable compounds that are orally absorbable. Meanwhile, since the use of the ADME model to predict the potential compounds of HCC has limitations [33], in order to avoid the omission of potential compounds, we searched a large number of references and included oral absorbable compounds with bioactivity.

Finally, combined with reference [34–42] and HPLC results, a total of 176 compounds were included: salvianolic acid B, rosmarinic acid, neocryptotanshinone, salvilenone, hirudin, glycyrrhizin, licochalcone G, isoimperatorin, salviolone, 66277-20-1, isotanshinone II, 537-15-5, prolithospermic acid, przewalskin A, sugiol, miltionone I, glabridin, dihydrotanshinone I, (E)-3-[2-(3,4-dihydroxyphenyl)-7-hydroxy-benzofuran-4-yl]acrylic acid (MOL007048), mairin, 3-beta-hydroxymethyllenetanshiquinone, arachidonate, girinimbin, fumarine, 97399-70-7, NSC122421, salvilenone I, tanshindiol B, poricoic acid B, isodalbergin, cycloartenol, poncimarin, poriferasterol, sitosterol, arachidonic acid, inermin, 1,2,5,6-tetrahydrotanshinone, 5-hydroxymethylfurfural, licochalcone B, bifendate, (Z)-3-[2-[(E)-2-(3,4-dihydroxyphenyl)vinyl]-3,4-dihydroxyphenyl]acrylic acid (MOL007140), ononin, diop, hirudonucleodisulfide A, formononetin, (6S)-6-(hydroxymethyl)-1,6-dimethyl-8,9-dihydro-7H-naphtho[8,7-g]benzofuran-10,11-dione (MOL007155), catalpol, dehydroeburicoic acid, suchilactone, dehydrotanshinone IIA, MOL000273, ent-epicatechin, ginsenoside Rg5, dan-shexinkum D, stigmasterol, trametenolic acid, chrysanthemaxanthin, deoxyharringtonine, isovitexin, quercetin, 11,14-eicosadienoic acid, isoliquiritigenin, o-nornuciferine, isolicoflavonol, przewalskin B, stachyose, beta-sitosterol, dihydrotanshinlactone, tanshinone IIA, eriodyctiol (flavanone), prangenin hydrate, rehmannioside A, celabenzine, licoisoflavone B, vestitol, 5z-eicosenoic acid, danshenol A, santalol, malkangunin, prangenin, tanshinaldehyde, licoricone, 520-26-3, miltirone, poriferast-5-en-3beta-ol, gomisin B, naringenin, obacunone, deoxyneocryptotanshinone, panaxadiol, przewaquinone F, 1,7-dihydroxy-3,9-dimethoxy pterocarpene, 4-methylenemiltirone, calycosin, (6S,7R)-6,7-dihydroxy-1,6-dimethyl-8,9-dihydro-7H-naphtho[8,7-g]benzofuran-10,11-dione (MOL007070), licochalcone A, 97411-46-6, poricoic acid C, *α*-amyrin, MOL000287, frutinone A, hirudonucleodisulfide B, didymin, dihomolinolenic acid, nuciferin, ginsenoside Rh4, C09092, aposiopolamine, microstegiol, sclareol, formyltanshinone, MOL000280, alexandrin, 73340-41-7, 87112-49-0, ergosterol peroxide, dianthramine, danshenspiroketallactone, manool, ergosta-7,22E-dien-3beta-ol, methylenetanshinquinone, (+)-dehydrodiconiferyl alcohol, 64997-52-0, epidanshenspiroketallactone, przewaquinone E, isoponcimarin, 7,9(11)-dehydropachymic acid, 3,9-di-O-methylnissolin, spinasterol, rehmannioside D, 2-(4-hydroxy-3-methoxyphenyl)-5-(3-hydroxypropyl)-7-methoxy-3-benzofurancarboxaldehyde (MOL007050), miltirone II, 7-O-methylisomucronulatol, ammidin, armepavine, acteoside, remerin, 5,7,4′-trimethylapigenin, hederagenin, neohesperidin, danshenol B, 1-methoxyphaseollidin, (-)-catechin, luteolin, MOL000285, isosinensetin, isocryptotanshinone, salvianolic acid G, glabrene, 64474-51-7, 3*α*-hydroxytanshinone IIA, sinensetin, schottenol, vitexin, glycyrin, tetramethoxyluteolin, cryptotanshinone, neocryptotanshinone II, przewaquinone B, przewaquinone C, nobiletin, miltipolone, tanshinone VI, cerevisterol, calycosin 7-O-glucoside, astragaloside IV, poricoic acid A, isorhamnetin, 6-methoxy aurapten, kaempferol, pachymic acid, machiline, glycyrrhetinic acid, miltionone II, 5,6-dihydroxy-7-isopropyl-1,1-dimethyl-2,3-dihydrophenanthren-4-one (MOL007036), and jaranol.

### 2.3. HDC Potential Target Prediction and DCM Gene Collection

SciFinder (http://scifinder.cas.org) and PubChem (https://pubchem.ncbi.nlm.nih.gov/) are used to retrieve and collect the structures of each potential compound. ChemBioDraw 14 is used to draw the structure of compounds and save it in mol2 format. The mol2 format files were input into PharmMapper (http://lilab-ecust.cn/pharmmapper/) for target prediction [43]. The UniProtKB (http://www.uniprot.org/) database is used to correct the target source species as *Homo sapiens*, and the target name is corrected to the official symbol. The details are described in Table S1 (see Supplementary Material). After that, “Diabetic cardiomyopathy” is used as a search term to search for DCM-related genes in GeneCards (http://www.genecards.org/) [44] and the OMIM (https://www.omim.org/) database [45].

### 2.4. Network Construction and Analysis Methods

HDC potential targets and DCM genes were introduced into STRING 11.0 (http://string-db.org/) to collect protein-protein interaction (PPI) data [46]. First, we selected the “Multiple proteins” tool and limited the species to “*Homo sapiens*”, then we saved the TSV format file of the PPI data. The node1, node2, and combined score information in the TSV file is imported into Cytoscape 3.7.1 software to build a PPI network [47]. Additionally, the Database for Annotation, Visualization, and Integrated Discovery (DAVID) ver 6.8 (https://david-d.ncifcrf.gov) was utilized to perform Gene Ontology (GO) enrichment analysis and pathway enrichment analysis [48].

### 2.5. Experimental Materials

#### 2.5.1. Experimental Animal

Eight 8–9-week-old specific pathogen-free- (SPF-) grade male Wistar rats and 42-week-old SPF-grade male GK rats with a body weight of 200 ± 20 g were purchased from Shanghai SLAC Laboratory Animal Co., Ltd. (certificate number: 311615200002581). Adapted feeding was provided for 1 week. Wistar rats were fed with ordinary feed, and GK rats were fed with high-sugar and high-fat diet. The formula of high-sugar and high-fat feed is as follows: basic feed—60.7%, sucrose—15%, cholesterol—4%, bile salt—0.3%, lard—10%, and egg yolk powder—10% (feed source: Hunan SLAC Laboratory Animal Co., Ltd.). All animals' care and experimental procedures were approved by the Animal Ethics Committee of Hunan University of Chinese Medicine and were in accordance with the National Institute of Health's Guide for the Care and Use of Laboratory Animals (approval number: 2014-19).

#### 2.5.2. Experimental Drugs

*(1) HDC*. *Licorice* (6 g), *Hedysarum multijugum* Maxim. (20 g), *Santalum album* L. (2 g), *Aurantii Fructus Immaturus* (10 g), *Platycladi Semen* (10 g), *Radix Salviae* (15 g), *Trichosanthis Radix* (12 g), *hirudo* (3 g), *Folium Nelumbinis* (15 g), *Rehmanniae Radix Praeparata* (15 g), *Panax ginseng* C. A. Mey. (9 g), and *Poria cocos* (Schw.) Wolf (8 g) were purchased from the First Affiliated Hospital of Hunan University of Chinese Medicine. It was identified by Professor Xia Xinhua, School of Pharmacy, Hunan University of Traditional Chinese Medicine, and the voucher specimens were deposited in the Herbarium of Hunan University of Chinese Medicine (voucher number: 2015067). HDC was cooked with water and concentrated to 4 g/mL of crude drug. Glipizide sustained-release tablets were purchased from Shi Weiya (Tianjin) Pharmaceutical Co., Ltd. (specification: 30 mg/tablet × 30 tablets), dissolved in physiological saline, and administered to rats at 2.7 mg/kg.

#### 2.5.3. Reagents and Instruments

The following were used: superoxide dismutase (SOD) kit, malondialdehyde (MDA) kit, and glutathione peroxidase (GSH-Px) kit (Nanjing Jiancheng Biotechnology Research Institute; A001-1-1-48T, A003-1-48T, and A005-48T); Bax and Bcl-2 rabbit anti-mouse polyclonal antibody (Proteintech, 50599-2-Ig; 12789-1-ap); and a full automatic microplate reader (PW-812) and multifunctional microplate analyzer (MB-530) (Shenzhen Huisong Technology Development Co., Ltd.).

### 2.6. Experimental Methods

#### 2.6.1. Animal Grouping and Intervention

Wistar rats were in the blank group and fed with ordinary feed. Forty-two GK rats were fed with a high-sugar and high-fat diet for 4 weeks. Blood was collected from the tail vein to measure fasting plasma glucose (FPG) and random blood glucose. Rats that presented twice withFPGs ≥ 7.0 mmol/Lorrandom blood glucose ≥ 11.1 mmol/L were considered successful models. Subsequently, 40 GK rats that were successfully modeled were divided into 5 groups according to the random number table: (1) the model group, (2) the gliclazide group, (3) the HDC low-dose group, (4) the HDC middle-dose group, and (5) the HDC high-dose group. Rats in each group were given the corresponding drugs for intragastric administration. The model group and the blank group were fed with an equal volume of distilled water. The HDC low-, medium-, and high-dose groups were administered at 6, 12, and 24 g/kg, respectively, and the gliclazide group was administered at 2.7 mg/kg, with an intragastric volume of 10 mL/kg. Rats in each group were administrated once a day for 10 weeks. During the experiment, one rat in the HDC low-dose group and one in the middle-dose group died, one of whom died of airway injury, and the other died of unknown cause.

#### 2.6.2. Indicator Detection

The body weight of each rat was recorded, and blood was collected from the tail vein to measure FPG. After the last administration, the rats were fasted overnight for 12 hours. The weight of each group of rats was weighed before injection, and the rats in each group were injected with 10% chloral hydrate at a body weight of 3 mL/kg. Blood was taken through the abdominal aorta and centrifuged at 3500 r/min at 4°C for 10 min, and the supernatant was taken. Glycated hemoglobin (HbA1c) was detected using a fully automatic biochemical analyzer. The sera SOD, MDA, and GSH-Px were tested strictly according to the kit instructions. The left ventricular myocardial tissue was taken, fixed in 4% paraformaldehyde solution, embedded in paraffin, and observed with pathological morphology such as HE staining and PAS staining. The expression of Bax and Bcl-2 was detected by immunohistochemistry.

### 2.7. Statistical Analysis

All measurement data are expressed as “*x* ± *s*,” and data are processed using SPSS 23.0 statistical software. One-way analysis of variance was used for pairwise comparison between multiple groups. The LSD test is used when the variance is uniform, and Dunnett's T3 test is used when the variance is uneven. A difference of *P* < 0.05 was considered to be statistically significant.

## 3. Results

### 3.1. HDC Potential Targets and DCM Genes

**Figure 2:**
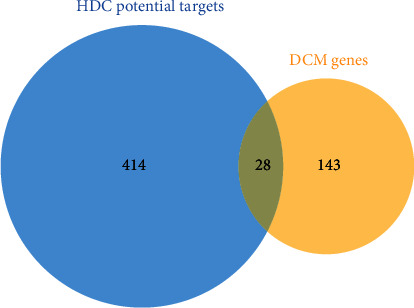
Venn diagram of HDC potential targets and DCM genes.

A total of 442 HDC potential targets were obtained. Among them, *Platycladi Semen* gets 305 potential targets, *Radix Salviae* includes 435 potential targets, *Rehmanniae Radix Praeparata* gets 322 potential targets, *Poria cocos(Schw.) Wolf.* includes 235 potential targets, *licorice* gets 306 potential targets, *Folium Nelumbinis* gets 399 potential targets, *Hedysarum multijugum* Maxim. includes 405 potential targets, *Panax ginseng* C. A. Mey. gets 345 potential targets, *hirudo* includes 382 potential targets, *Santalum album* L. gets 396 potential targets, *Trichosanthis Radix* includes 234 potential targets, and *Aurantii Fructus Immaturus* gets 406 potential targets. Meanwhile, a total of 171 DCM-related genes were obtained from the OMIM and GeneCards databases. There are overlapping targets between HDC potential targets and the DCM genes (Figure 2).

### 3.2. HDC-DCM PPI Network Analysis

#### 3.2.1. HDC-DCM PPI Network Construction

**Figure 3:**
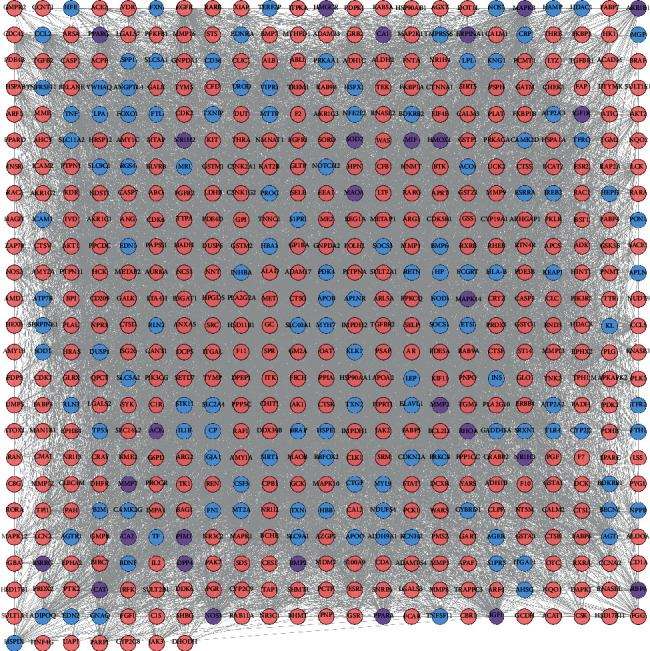
HDC-DCM PPI network (blue, pink, and purple circles stand for DCM genes, HDC potential targets, and HDC-DCM targets, respectively).

The HDC potential targets, DCM genes, and PPI data were input into Cytoscape 3.7.1 to construct the HDC-DCM PPI network. This network is composed of 559 nodes (402 HDC potential target nodes, 129 DCM gene nodes, and 28 HDC-DCM target nodes) and 9767 edges (Figure 3).

#### 3.2.2. Biological Processes of the HDC-DCM PPI Network

**Figure 4:**
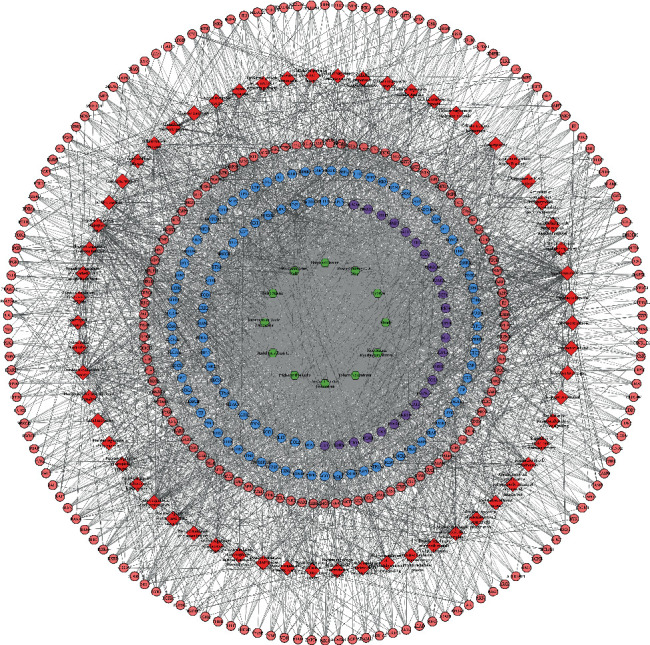
Biological processes of the HDC-DCM PPI network (blue, pink, and purple circles stand for DCM genes, HDC potential targets, and HDC-DCM targets, respectively; green hexagons stand for herbs; red diamonds stand for biological processes).

**Figure 5:**
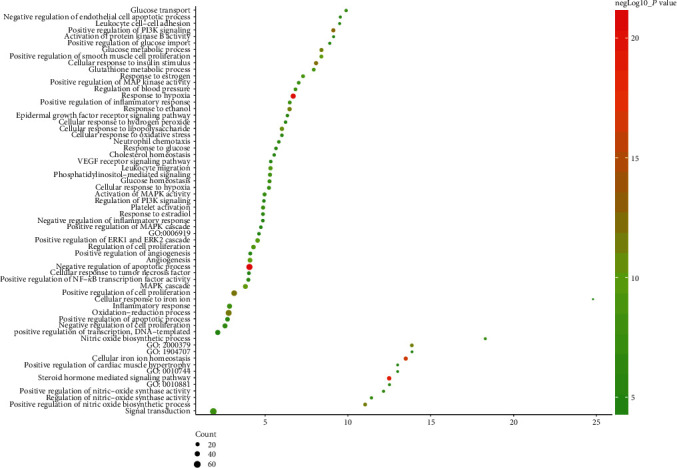
Bubble chart of biological processes (*X*-axis stands for fold enrichment).

The HDC potential targets and DCM genes were input into DAVID for GO enrichment analysis, and 62 DCM-related biological processes were returned (Figure 4). These biological processes are arranged in descending order of enrichment (negatively correlated with *P* value) and count. The top 10 biological processes are as follows: negative regulation of apoptotic process (*P* = 1.84∗10^−21^, count = 63), response to hypoxia (*P* = 7.58∗10^−21^, count = 39), steroid hormone-mediated signaling pathway (*P* = 1.36∗10^−19^, count = 24), cellular iron ion homeostasis (*P* = 4.41∗10^−17^, count = 20), positive regulation of phosphatidylinositol 3-kinase signaling (*P* = 2.09∗10^−13^, count = 20), cellular response to insulin stimulus (*P* = 5.73∗10^−13^, count = 21), positive regulation of cell proliferation (*P* = 1.22∗10^−12^, count = 50), oxidation-reduction process (*P* = 2.19∗10^−12^, count = 57), positive regulation of nitric oxide biosynthetic process (*P* = 4.03∗10^−12^, count = 16), and positive regulation of reactive oxygen species metabolic process (*P* = 4.19∗10^−12^, count = 14) (Figure 5) (see Table S3).

#### 3.2.3. Signaling Pathways of the HDC-DCM PPI Network

**Figure 6:**
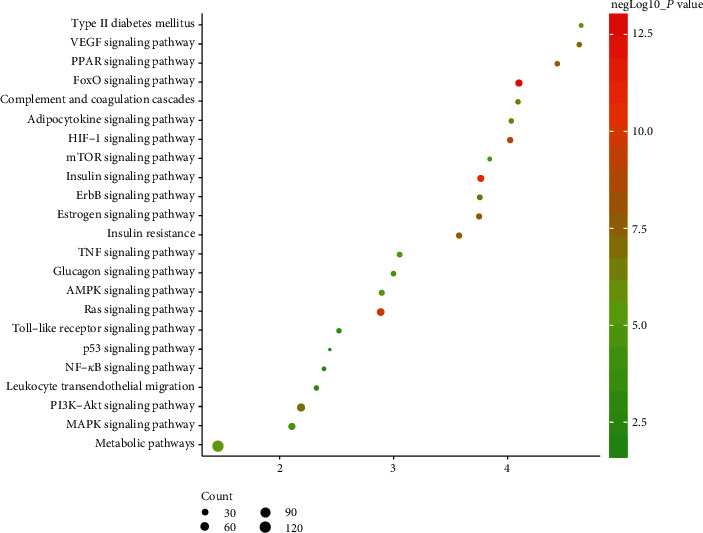
Bubble chart of signaling pathways (*X*-axis stands for fold enrichment).

All HDC potential targets and DCM genes were introduced into DAVID for pathway enrichment analysis. Eventually, a total of 23 DCM-related signaling pathways were returned. These signaling pathways are involved in multiple aspects of DCM's treatment. The bubble chart is drawn based on the *P* value of the signaling pathway, fold enrichment, and the number of genes included (Figure 6 and Table S4).

### 3.3. Effect of HDC on the General Condition of GK Rats

The rats in the blank group had white, clean, shiny hair. They were in a good state of mind and were active. The litter was clean and odorless. Their body weights were significantly increased, and the tongue and lip colors were red and moist. The rats of the model group had yellow, dull, and messy hair, which fell easily. They were apathetic; had significantly reduced activity; were sluggish, drowsy, and sloppy; and kept on crowding. The litter was moist and had a bad smell. Their stool was soft, and their tongues and lips were dark purple. The general condition of the HDC low-dose group did not improve significantly. The hairs of the rats in the gliclazide group and HDC medium- and high-dose groups were not significantly yellowed. Their mental state and activity were good, and their lip and tongue colors were ruddy.

### 3.4. Effect of HDC on FPG and HbA1c in GK Rats

After 10 weeks of drug intervention, compared with the blank group, FPG in the model group was significantly increased (*P* < 0.01). Compared with the model group, FPG in the HDC low-dose group decreased (*P* < 0.05); FPG in the gliclazide group and the HDC medium- and high-dose groups decreased significantly (*P* < 0.01) (Table 1).

Compared with the blank group, HbA1c in the model group was significantly increased (*P* < 0.01). Compared with the model group, HbA1c in the gliclazide group was significantly reduced (*P* < 0.01), HbA1c in the HDC high-dose group was reduced (*P* < 0.05), and there was no significant difference in HbA1c of the HDC low- and middle-dose groups (*P* > 0.05) (Table 1).

### 3.5. Effect of HDC on Oxidative Stress-Related Indexes in GK Rats

Compared with the blank group, SOD and GSH-Px were significantly reduced (*P* < 0.01) and MDA was significantly increased (*P* < 0.01) in the model group. Compared with the model group, SOD and GSH-Px increased significantly (*P* < 0.01) and MDA decreased (*P* < 0.05) in the gliclazide group and the HDC high-dose group. Compared with the model group, GSH-Px in the HDC medium- and low-dose groups was significantly increased (*P* < 0.01 or *P* < 0.05); there was no significant difference in MDA and SOD of the HDC medium- and low-dose groups (*P* > 0.05) (Table 2).

### 3.6. Effect of HDC on Myocardial Pathomorphology in GK Rats

#### 3.6.1. HE Staining

**Figure 7:**
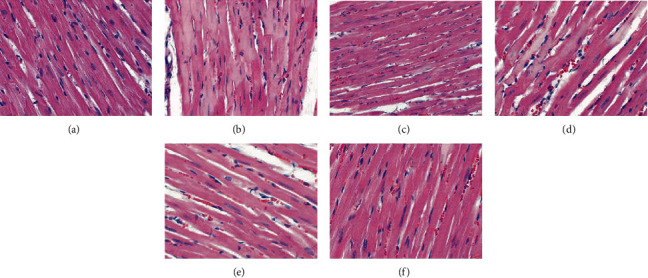
Pathological changes of myocardial tissue in rats (HE staining, ×400; (a) blank group, (b) model group, (c) gliclazide group, (d) HDC low-dose group, (e) HDC medium-dose group, and (f) HDC low-dose group).

Under light microscopy, the nucleus of the myocardial cells in the blank group was round or oval. The nucleus was arranged in the central cell, and the myofibrils were arranged neatly. The myocardial fibers were not broken and rearranged. In the model group, the nucleus edges of myocardial cells were blurred, with burrs. Myocardial fibers are disorderly arranged, showing broken and incomplete fibers. Myocardial cells are hypertrophic, edema is obvious (light stain), and myocardial cells with shrinkage and degeneration are visible. In the gliclazide group, the myocardial fibers are arranged neatly, with occasional lysis and rupture, and myocardial cell edema and necrosis are significantly reduced. In the HDC low-dose group, myocardial fibers showed obvious lysis and rupture, and myocardial cells showed edema, necrosis, and disordered arrangement. In the HDC and high-dose groups, myocardial myofibrils were arranged neatly, the edges were smooth, there were no obvious breaks, and no obvious edema and necrotic cells were seen (Figure 7).

#### 3.6.2. PAS Staining

**Figure 8:**
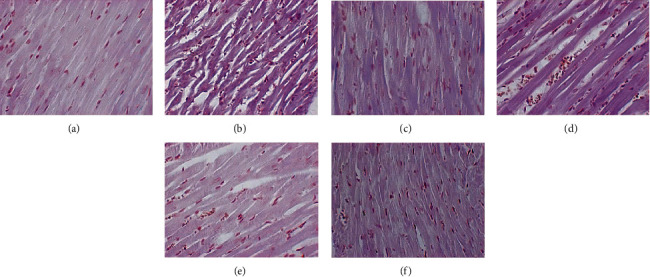
Glycogen deposition results in rat myocardial tissue (PAS staining, ×400; (a) blank group, (b) model group, (c) gliclazide group, (d) HDC low-dose group, (e) HDC medium-dose group, and (f) HDC low-dose group).

The myocardial tissue of the blank group was lightly stained, and there were very few purple-red glycogen-positive staining areas. A large amount of deeply stained glycogen-positive material was clearly visible in the myocardial tissue of the model group. In the HDC low-dose group, the glycogen-positive substances were significantly deposited in the myocardial tissue. In the gliclazide group and HDC medium- and high-dose groups, myocardial tissue was lightly stained, and glycogen-positive substances were significantly reduced (Figure 8).

### 3.7. Effect of HDC on Bax and Bcl-2 Protein Expression in Myocardium

**Figure 9:**
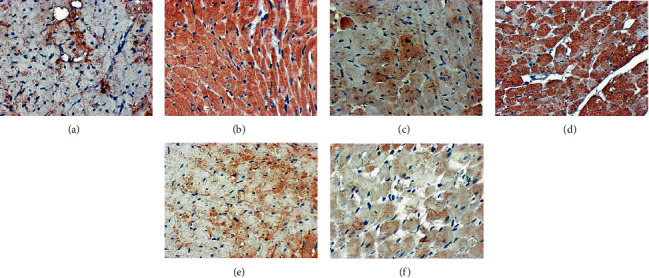
Bax protein expression in rat myocardial tissue (×400; (a) blank group, (b) model group, (c) gliclazide group, (d) HDC low-dose group, (e) HDC medium-dose group, and (f) HDC low-dose group).

The Bax protein in the myocardial tissues of the blank group was lightly stained, weakly expressed, and minimal in thickness. Compared with the blank group, Bax expression in the model group increased significantly (*P* < 0.05). Compared with the model group, the expression of Bax in the gliclazide group and HDC high-dose group was significantly reduced (*P* < 0.01); the expression of Bax in the HDC medium-dose group was reduced (*P* < 0.05) (Table 3 and Figure 9).

**Figure 10:**
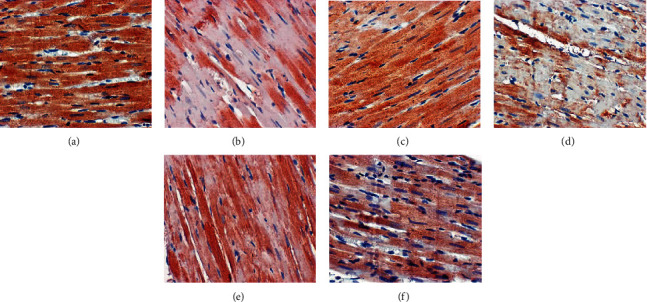
Bcl-2 protein expression in rat myocardial tissue (×400; (a) blank group, (b) model group, (c) gliclazide group, (d) HDC low-dose group, (e) HDC medium-dose group, and (f) HDC low-dose group).

The staining of Bcl-2 protein in the myocardial tissue of the blank group was dark brown with strong positive expression and moderate thickness. Compared with the blank group, the expression of myocardial Bcl-2 in the model group was significantly reduced (*P* < 0.01). Compared with the model group, the expression of Bcl-2 in the gliclazide group and HDC medium- and high-dose groups increased significantly (*P* < 0.01); there was no significant difference in the Bcl-2 expression in the HDC low-dose group (*P* > 0.05) (Table 3 and Figure 10).

## 4. Discussion

### 4.1. Discussion for HDC Potential Components and Targets

Modern pharmacological research divides the effective compounds of *Radix Salviae* into water-soluble and fat-soluble compounds. Fat-soluble compounds are mainly tanshinones, and water-soluble compounds are mainly tanshinins [49]. *Radix Salviae* can resist endothelial cell damage caused by free radicals through antioxidant effects, increase superoxide dismutase (SOD) content, and downregulate malondialdehyde (MDA). It can also regulate vasoconstriction and relaxation, maintain the dynamic balance of endothelin-1 (ET-1) and nitric oxide (NO), and inhibit the expression of inflammatory factors such as adhesion factors, thereby regulating vascular endothelial cell function, inhibiting blood cell adhesion, and preventing thrombosis [50–53]. Certain monomers of *Radix Salviae* can also inhibit endothelial cells by downregulating caspase-3, an important member of the apoptotic signaling pathway [54]. Recent studies have also shown that *Radix Salviae* injection can improve cardiac function in diabetic rats and prevent myocardial tissue from downregulating TSP-1 and TGF-*β*1 [55].

High expression of angiotensin II type 2 receptor (AT2) may accelerate the apoptosis of cardiomyocytes in diabetic patients, and *Hedysarum multijugum* Maxim. can protect diabetic rats from developing DCM by downregulating AT2 [56]. Studies show that the astragalus decoction can reduce the expression of Nrf2 by regulating the ACE2/AngII-AT1R-NADPH oxidase pathway and participate in the myocardial protection of diabetes, but its hypoglycemic effect is not obvious [57]. Astragaloside IV can reduce oxidative stress and delay myocardial hypertrophy by inhibiting the AMPK/mTOR pathway, thereby improving myocardial damage [58]. Cao et al. found that (1) astragaloside IV can downregulate blood glucose levels in diabetic rats, improve their myocardial structure, and enhance cardiac function; (2) astragaloside IV can promote the mitochondrial biosynthesis of myocardial cells in diabetic rats through the PGC-1*α* pathway, so as to improve myocardial energy metabolism; and (3) astragaloside IV can downregulate the expression of apoptotic proteins through the PGC-1*α* pathway and effectively inhibit myocardial cell apoptosis in diabetic rats [59]. The results of in vitro cell experiments indicate that astragaloside IV has a protective effect on cardiomyocyte hypertrophy induced by high glucose in neonatal rats, which may be related to inhibiting calcium overload and reducing CaN protein expression [60].

### 4.2. Discussion for Biological Processes of HDC-DCM PPI Network

Current research indicates that abnormal blood glucose, abnormal blood lipids, left ventricular hypertrophy, metabolic abnormalities, endothelial dysfunction, changes in extracellular matrix of vascular calcification and hypercoagulability, small vascular disease, cardiac autonomic neuropathy, insulin resistance, oxidative stress, and cell metabolism are all important promoters, which are involved in the occurrence and development of DCM [61, 62]. For example, advanced glycation end products (AGEs) can bind to AGE receptors on cardiac cell membranes, thereby promoting myocardial fibrosis and increasing expression of oxidative stress mediators [63]. Meanwhile, hyperglycemia activates the renin-angiotensin-aldosterone system (RAAS). The increase of angiotensin II (Ang II) stimulates the proliferation of myocardial fibroblasts and changes in collagen anabolic metabolism through cell surface angiotensin receptor 1, causing fibrosis of myocardial interstitial and surrounding blood vessels. Ang II can also stimulate myocardial cell proliferation and myocardial cell hypertrophy, cause diastolic dysfunction, and eventually lead to myocardial hypertrophy and myocardial fibrosis [64, 65]. A hyperglycemic environment can cause increased electron donor generation in the mitochondrial tricarboxylic acid cycle. When the mitochondrial membrane potential rises beyond a certain range, a large number of reactive oxygen species will be generated, and oxidative stress will increase; at the same time, the gene expression of antioxidant enzymes such as SOD and catalase is downregulated, and the active groups are glycosylated, causing the body's antioxidant capacity to decrease. This eventually leads to the accumulation of a large number of reactive oxygen species, causing damage to the structure and composition of myocardial cells [66, 67].

Insulin resistance is an early metabolic abnormality of type 2 diabetes (T2DM) [68], which results in hyperinsulinemia due to decreased insulin reactivity. Hyperinsulinemia can cause myocardial cell hypertrophy and myocardial fibrosis through various mechanisms and can also cause epigenetic and genetic changes. Activation of multiple transcription factors leads to the deposition of extracellular matrix proteins and myocardial cell hypertrophy, which promote the occurrence and development of DCM [69–71]. In addition, insulin resistance can promote the oxidation of nonesterified fatty acids in the myocardial matrix; excessive intake of fatty acids will exceed the oxidative capacity of cardiomyocyte mitochondria, and ectopic lipid deposition in the heart will induce lipotoxicity to the heart [72]. These lipid metabolism toxicants can interfere with normal cellular signals; result in myocardial cell apoptosis, cell damage, and mitochondrial dysfunction; and ultimately lead to reduced cardiac contractile function [73, 74]. Therefore, abnormal cardiac fat metabolism is an important cause of DCM [75, 76].

In addition, new research shows that calcium homeostasis is also an important part of DCM. In the heart of T2DM mice, intracellular free Ca^2+^ is increased, intracellular Ca^2+^ decay time is prolonged, Ca^2+^ transients are slowed down, sarcoplasmic reticulum calcium pump is reduced, and Ca^2+^ reuptake is impaired [77]. Inflammatory factor-mediated biological processes also play a huge role in DCM [78], such as tumor necrosis factor-*α* (TNF-*α*), interleukin- (IL-) 6, IL-8, and monocyte chemotactic protein 1. These cytokines affect the population of cells that contribute to the rational remodeling and oxidative stress of heart disease, including cardiac muscle cells, endothelial cells, fibroblasts, and smooth muscle cells [79–81].

In short, DCM is associated with chronic hyperglycemia-related metabolism, insulin resistance, lipotoxicity, calcium homeostasis, and RAAS. Our research also shows that HDC can interfere with blood pressure regulation, glucose metabolism regulation, inflammation regulation, vascular endothelial function, insulin resistance, myocardial dysfunction and myocardial hypertrophy, oxidative stress, and other related biological processes.

### 4.3. Discussion for Signaling Pathways of HDC-DCM PPI Network

The metabolic environments related to T2DM are hyperglycemia, which can increase the levels of circulating fatty acids and triglycerides and cause abnormal glucose and lipid metabolism and lipid accumulation in myocardial cells, and hyperinsulinemia, which can enhance the expression of inflammatory cytokines, change the molecular signaling pathways in multiple myocardial cells, and damage the contraction of the heart. The signaling pathways related to these factors play an important role in DCM, such as the cardiac ubiquitin proteasome system, the FoxO transcription factor signaling pathway, the hexosamine biosynthetic pathway, the polyol pathway, protein kinase C signaling, NF-*κ*B signaling, peroxisome proliferator-activated receptor signaling, Nrf2 pathways, the mitogen-activated protein kinase pathways, and microRNAs, especially the AMPK signaling pathway [82]. Since blood glucose levels, fatty acid oxidation, and glycogen metabolism are regulated by AMPK, AMPK plays a vital role in the formation of DCM. Activated AMPK can not only improve diabetic myocardial damage, reduce fatty acid synthesis, increase glucose intake and metabolism, and reduce liver glucose production but also regulate mitochondrial function and cell proliferation. Recently, the small-molecule AMPK-directed agonist CNX-012-570, which can regulate insulin and sugar ester metabolism levels, has entered the preclinical research stage [83].

Our research shows that HDC can regulate the FoxO signaling pathway, the insulin signaling pathway, the Ras signaling pathway, the HIF-1 signaling pathway, the estrogen signaling pathway, insulin resistance, the PPAR signaling pathway, the VEGF signaling pathway, the PI3K-Akt signaling pathway, the ErbB signaling pathway, the complement and coagulation cascades, the adipocytokine signaling pathway, type II diabetes mellitus, the metabolic pathways, the AMPK signaling pathway, the TNF signaling pathway, the mTOR signaling pathway, the glucagon signaling pathway, the MAPK signaling pathway, the Toll-like receptor signaling pathway, and so on.

### 4.4. Discussion for Animal Experiments

Animal experiments used spontaneous nonobese T2DM GK rats combined with a high-sugar and high-fat diet to establish the DCM model. GK rats are derived from Wistar rats whose oral glucose tolerance is at the upper limit after repeated inbred generations. This rat species mainly manifests increased fasting blood glucose, high blood glucose after eating, impaired insulin secretion, and glucose intolerance, which is why it is one of the ideal internationally recognized animal models for studying T2DM, and is easy to complicate with cardiovascular disease after long-term illness [84, 85]. The results of this study show that GK rats have a slow increase in body weight in the late experimental period, which is related to the nonobese nature of the rat species. The FPG in the model group was significantly higher than that in the blank group. HE staining showed hypertrophy of cardiomyocytes, disordered arrangement, breakage, and focal necrosis of myocardial fibers, while PAS staining showed pathological changes such as the deposition of a large amount of glycogen-positive substances in myocardial tissue. This indicates that the DCM model was successfully constructed in this study, and the molding rate was 95.2%. The positive control drug, gliclazide sustained-release tablet, is the second-generation sulfonylurea oral hypoglycemic agent, and it is one of the first-choice drugs in clinical treatment of T2DM with nonobesity, normal or low fasting insulin levels. Its structure contains a nitrogen heterocyclic ring, which has the function of scavenging free radicals; therefore, it has the unique effect of improving oxidative stress [86, 87], and it can reduce the incidence of atherosclerosis and cardiovascular disease [86, 88]. Oxidative stress is a common ground for insulin resistance and the onset of diabetes and cardiovascular disease [89]. SOD, GSH-Px, and so on are important barriers for the body to resist oxidative damage, and SOD is the only natural antioxidant enzyme in the body that can remove superoxide anions and plays an important role in combating oxidative damage [89]. GSH-Px is an important peroxide-degrading enzyme widely present in the body. It can catalyze reduced glutathione (GSH) into oxidized glutathione (GSSG) and protect cells from the interference and damage of peroxide. MDA is an oxidative end product of free radicals acting on peroxidation of lipids and is cytotoxic. This experimental study shows that the levels of SOD and GSH-Px in the model group are significantly reduced and the levels of MDA are significantly increased, while HDC can significantly increase the levels of SOD and GSH-Px and reduce the content of MDA, indicating that HDC has a good antioxidant effect. Bcl-2 is the first gene to be found to be involved in apoptosis. The Bcl-2 gene family includes Bcl-2 and other apoptosis-inhibiting proteins and Bax and other proapoptotic proteins. A large number of studies have found that oxidative stress mediates myocardial cell apoptosis, and a large amount of ROS production under hyperglycemia can activate multiple signaling pathways to induce cell autophagy, damage endothelial cells, and cause myocardial hypertrophy [90]. In addition, ROS can also cause mitochondrial damage, release proapoptotic genes, cause a series of cascade reactions to activate the downstream caspase family, and initiate the apoptotic process of cells, thereby causing cardiomyocyte damage [91]. In this study, we found that Bcl-2 expression was significantly reduced and Bax expression was significantly increased in the model group, while HDC significantly increased Bcl-2 expression and decreased Bax expression, indicating that HDC has the effect of inhibiting myocardial cell apoptosis, the mechanism of which may be to reduce the damage of cardiomyocytes by enhancing the antioxidant capacity.

The limitation of this study is that we only conducted research on HDC antioxidant capacity and antiapoptosis in rats. Due to the difficulty of obtaining human myocardial tissue, we have not conducted clinical trials for the time being, which means that the clinical transformation capabilities of this research need to be strengthened. Therefore, in the future, we will further carry out clinical randomized controlled studies, using mononuclear cells (PBMC) to explore and verify the mechanism of HDC at the aspects of oxidative stress and inflammation.

TCM has great advantages in preventing and treating complications of diabetes. HDC can play a multitarget and multidirectional treatment effect on the disease, which is also the characteristics and advantages of the herbal medicine. However, the internal molecular mechanism of its function is a complex network system, and there are still many places worthy of further discussion. Meanwhile, it may be one of the treatment strategies to delay the development of DCM by enhancing the body's antioxidant defense system clearance ability and inhibiting the damage of oxidative stress to the myocardium.

## 5. Conclusion

HDC improves DCM through its antiapoptotic and anti-inflammatory effects. HDC may play a therapeutic role by improving cardiomyocyte apoptosis in DCM rats.

## Figures and Tables

**Table 1:** FPG and HbA1c in each group (*x* ± *s*).

Group	*n*	FPG (mmol/L)	HbA1c (%)
Blank	8	4.14 ± 0.34	3.97 ± 0.28
Model	8	9.56 ± 2.86^∗∗^	6.49 ± 0.71^∗∗^
Gliclazide	8	4.93 ± 0.44^##^	5.37 ± 0.47^##^
HDC low dose	7	7.21 ± 0.64^#^	6.20 ± 0.82
HDC medium dose	7	5.51 ± 0.32^##^	6.06 ± 0.94
HDC high dose	8	4.98 ± 0.69^##^	5.42 ± 0.44^#^

Compared with the blank group: ^∗∗^*P* < 0.01. Compared with the model group: ^#^*P* < 0.05; ^##^*P* < 0.01.

**Table 2:** Oxidative stress-related indexes in each group (*x* ± *s*).

Group	*n*	SOD (U/mL)	GSH-Px (U/mL)	MDA (nmol/mL)
Blank	8	308.3 ± 39.73	842.5 ± 32.82	7.31 ± 1.20
Model	8	235.7 ± 52.15^∗∗^	757.7 ± 27.33^∗∗^	11.54 ± 2.29^∗∗^
Gliclazide	8	312.4 ± 28.63^##^	814.4 ± 46.98^##^	8.04 ± 1.74^#^
HDC low dose	7	278.9 ± 38.43	765.2 ± 23.73^#^	11.68 ± 3.19
HDC medium dose	7	279.5 ± 22.62	794.6 ± 33.12^##^	11.60 ± 2.26
HDC high dose	8	308.9 ± 45.00^##^	809.8 ± 25.95^##^	8.07 ± 2.46^#^

Compared with the blank group: ^∗∗^*P* < 0.01. Compared with the model group: ^#^*P* < 0.05; ^##^*P* < 0.01.

**Table 3:** Oxidative stress-related indexes in each group (*x* ± *s*).

Group	*n*	Bax (IOD)	Bcl-2 (IOD)
Blank	8	27.31 ± 11.44	116.40 ± 12.96
Model	8	132.00 ± 16.17^∗∗^	32.44 ± 6.49^∗∗^
Gliclazide	8	58.20 ± 17.15^##^	99.06 ± 31.83^##^
HDC low dose	7	103.30 ± 18.69	44.46 ± 9.33
HDC medium dose	7	76.18 ± 35.43^#^	95.07 ± 22.31^##^
HDC high dose	8	62.50 ± 22.61^##^	106.10 ± 14.85^##^

Compared with the blank group: ^∗∗^*P* < 0.01. Compared with the model group: ^#^*P* < 0.05; ^##^*P* < 0.01.

## Data Availability

The data used to support the findings of this study are included within the article and the supplementary information files.

## Supplementary Materials

Supplementary 1 Table S1: compound targets for each compound.

Supplementary 2 Table S2: diabetic cardiomyopathy genes.

Supplementary 3 Table S3: enrichment analysis of clusters based on Gene Ontology (GO) annotation of the HDC-DCM PPI network.

Supplementary 4 Table S4: pathway enrichment analysis of the HDC-DCM PPI network.
